# 567. Reassessment of a Piloted Enoxaparin Dosing Schedule for Venous Thromboembolism Prophylaxis in Burn Patients

**DOI:** 10.1093/jbcr/irag033.307

**Published:** 2026-04-06

**Authors:** Lauren A Lautenslager, Sam Stuart, Allison N Boyd, Todd A Walroth, Matthew Bjornstad, Alyssa Garelli, Leigh J Spera

**Affiliations:** Eskenazi Health, Indianapolis, IN; Indiana University School of Medicine, Indianapolis, IN; Eskenazi Health, Indianapolis, IN; Eskenazi Health, Indianapolis, IN; Eskenazi Health, Indianapolis, IN; Eskenazi Health, Indianapolis, IN; Indiana University/Eskenazi Health, Indianapolis, IN

## Abstract

**Introduction:**

Burn patients face a heightened risk of venous thromboembolism (VTE) secondary to endothelial injury, immobility, and systemic hypercoagulability, making prophylaxis essential. Standard prophylactic enoxaparin dosing often fails to achieve adequate anti-coagulation (measured as target anti-Xa levels) in this population. Our institution previously piloted a revised VTE prophylaxis protocol, evaluated in a small cohort. This study expands our analysis to a larger cohort to assess safety and efficacy.

**Methods:**

This single-center, retrospective cohort study compared a historical protocol group (3/1/21 - 5/31/21) with a new protocol group (7/1/22 - 8/31/22), with further analysis of additional new-protocol patients (11/20/23 - 3/2/2025). Adults with cutaneous/inhalation burns who received prophylactic enoxaparin with anti-Xa monitoring were included. Exclusions were non-burn admissions, frostbite injuries, and incarcerated status. The primary endpoint was protocol efficacy defined as percent of initial anti-Xa levels within goal range and time to goal anti-Xa level. Secondary endpoints evaluated were VTE incidence (inpatient/post-discharge), dosing interruptions with VTE, correlation to published enoxaparin dosing equations in burn patients, and bleeding events.

**Results:**

The median (IQR) initial anti-Xa level in the historical (n = 29) vs. new protocol group (n = 134) was 0.09 (0.04-0.14) units/mL vs. 0.22 (0.16-0.28) units/mL (*p*<.001). The median (IQR) time for patients to achieve goal anti-Xa level was 6 (3-8) days in the historical protocol and 3 (2-4) days in the new protocol group (*p*=.003). This improvement contrasts with our prior smaller analysis, in which time-to-target anti-Xa did not differ significantly (*p*=.456). Rates of inpatient VTE (*p*=.662), major bleeding (*p*=.068), and minor bleeding (*p*=.415) were comparable between groups. No other secondary outcomes differed significantly.

**Conclusions:**

Implementation of the revised protocol improved both initial anti-Xa levels and time to therapeutic range without increasing bleeding risk. The median anti-Xa value improved significantly from subtherapeutic to within goal range with the revisions to the protocol.

**Applicability of Research to Practice:**

This novel protocol more quickly and reliably achieves therapeutic levels of enoxaparin than previous protocols, without compromising safety. It can be replicated and utilized by other burn centers and warrants a multicenter analysis.

**Funding for the study:**

N/A.

